# Model pruning based on filter similarity for edge device deployment

**DOI:** 10.3389/fnbot.2023.1132679

**Published:** 2023-03-02

**Authors:** Tingting Wu, Chunhe Song, Peng Zeng

**Affiliations:** ^1^State Key Laboratory of Robotics, Shenyang Institute of Automation, Chinese Academy of Sciences, Shenyang, China; ^2^Key Laboratory of Networked Control Systems, Chinese Academy of Sciences, Shenyang, China; ^3^Institutes for Robotics and Intelligent Manufacturing, Chinese Academy of Sciences, Shenyang, China; ^4^University of Chinese Academy of Sciences, Beijing, China

**Keywords:** network acceleration, filter pruning, edge intelligence, network compression, convolutional neural networks

## Abstract

Filter pruning is widely used for inference acceleration and compatibility with off-the-shelf hardware devices. Some filter pruning methods have proposed various criteria to approximate the importance of filters, and then sort the filters globally or locally to prune the redundant parameters. However, the current criterion-based methods have problems: (1) parameters with smaller criterion values for extracting edge features are easily ignored, and (2) there is a strong correlation between different criteria, resulting in similar pruning structures. In this article, we propose a novel simple but effective pruning method based on filter similarity, which is used to evaluate the similarity between filters instead of the importance of a single filter. The proposed method first calculates the similarity of the filters pairwise in one convolutional layer and then obtains the similarity distribution. Finally, the filters with high similarity to others are deleted from the distribution or set to zero. In addition, the proposed algorithm does not need to specify the pruning rate for each layer, and only needs to set the desired FLOPs or parameter reduction to obtain the final compression model. We also provide iterative pruning strategies for hard pruning and soft pruning to satisfy the tradeoff requirements of accuracy and memory in different scenarios. Extensive experiments on various representative benchmark datasets across different network architectures demonstrate the effectiveness of our proposed method. For example, on CIFAR10, the proposed algorithm achieves 61.1% FLOPs reduction by removing 58.3% of the parameters, with no loss in Top-1 accuracy on ResNet-56; and reduces 53.05% FLOPs on ResNet-50 with only 0.29% Top-1 accuracy degradation on ILSVRC-2012.

## 1. Introduction

Deep neural networks(DNNs) have become one of the most widely used algorithms in image classification (Krizhevsky et al., 2012), object detection (Ren et al., 2015), video analysis (Graves et al., 2013), and other fields with far surpassing accuracy than traditional algorithms. However, the high computing power and memory requirements of DNNs make it difficult for edge devices to deploy them with low latency, low power consumption, and high precision (Uddin and Nilsson, 2020; Veeramanikandan et al., 2020; Zhang et al., 2020; Fortino et al., 2021). To address this problem, various methods have been proposed for network compression and inference acceleration, including lightweight architecture design (Howard et al., 2017; Zhang X. et al., 2018), network pruning (LeCun et al., 1990; Hassibi and Stork, 1993; Li et al., 2016), weight quantization (Courbariaux et al., 2015; Hubara et al., 2017), matrix factorization (Denton et al., 2014), and knowledge distillation (Hinton et al., 2015; Gou et al., 2021). Quantization compresses the model by reducing the size of the weights or activations. Matrix factorization is to approximate the large number of redundant filters of a layer using a linear combination of fewer filters. And knowledge distillation trains another simple network by using the output of a pre-trained complex network as a supervisory signal. Among them, network pruning compresses the existing network to reduce the requirements for space and computing power, to achieve real-time operation on portable devices. According to the granularity of pruning, network pruning methods can be divided into structured and unstructured pruning. Unstructured pruning requires specialized hardware and software for effective reasoning, and random connections will lead to poor cache locality and memory jump access, which makes acceleration very limited. Among structured pruning methods, filter pruning has received widespread attention because of its advantages of being directly compatible with current general-purpose hardware and highly efficient basic linear algebra subprogram (BLAS) libraries. The research in this paper belongs to the category of structured pruning, that is, the pruning granularity is at the level of convolution kernels.

Formally, for a CNN with weights of *W* and *L* convolutional layers, and *N*_*i*_ filters in each layer, determining which filter needs to be pruned is a combinatorial optimization problem, that can be expressed as follows (Zhou et al., 2019):


(1) $$\begin{array}{l} \{\begin{array}{l} \min_{ℳ}C(D;ℳ\circ W)\\ \min_{ℳ}\sum_{i=1}^{L}{\left\|{ℳ}_{i}\right\|}_{1} \end{array} \end{array}$$

where M is the mask of the filter, and C is the cost function of the CNN on dataset D. If there is a subset of convolution kernels such that the network can be pruned without performance degradation, it will be required to perform $2\overset{L}{\underset{i=1}{\sum }}N_{i}$ search and evaluation steps. For the current large network structure, this is an NP-hard problem, which is difficult to accurately solve by searching all possible subsets.

Among the simplest methods is the greedy method, or saliency-based method, which sorts weights by importance. The core problem is how to measure the importance of the filters. Recently a variety of filter pruning methods have been proposed to design more effective pruning guidelines. Hu et al. (2016) proposed using the average percentage of zero values (APoZ) to measure the importance of the activation value, which is defined as the proportion of zeroes in the activation values. Li et al. (2016) put forward a hypothesis based on the absolute value: the smaller the *l*_1_−*norm* of the filter is, the less its influence on the final result. Molchanov et al. (2016) utilized the absolute value of the first-order term in the expansion of the objective function relative to the activation function as the criterion for pruning. Liu et al. (2017) introduced a channel scaling factor to the BN layer, added *l*_1_ regularization to make it sparse, and then pruned the filters with a smaller scaling factor. He et al. (2019) developed a pruning method based on a geometric median to remove redundant filters.

Although the above works have achieved notable achievements, there are still many limitations: (1) Due to the different distributions of the values of the convolution kernels in different layers, the abovementioned pruning methods based on global or local criteria for sorting filters may ignore filters with smaller values in the sorting but extract edge features. Huang et al. (2020) compared different pruning standards and found that they have strong similarities, and that the importance of the obtained filters is almost the same, resulting in similar pruning structures. (2) Recent work (Liu et al., 2018) shows that the pruning structure is the key to determining the performance of the pruning model rather than the inheritance weight. Manually setting the pruning rate of each convolutional layer is equivalent to redesigning the network structure completely, and improper pruning rate settings will result in insufficient pruning or excessive pruning. In addition, for large networks, it is very expensive to accurately calculate the importance of the filters and set the pruning rate of each layer. (3) For special network structures such as residual blocks, most works only prune the channels of the middle layer of the block, which limits the space available for pruning. (4) The pruning process and the large number of fine-tuning required to restore the pruning performance lead to an excessively long pruning cycle, which is also the direction that needs to be optimized at present.

**Figure 1:**
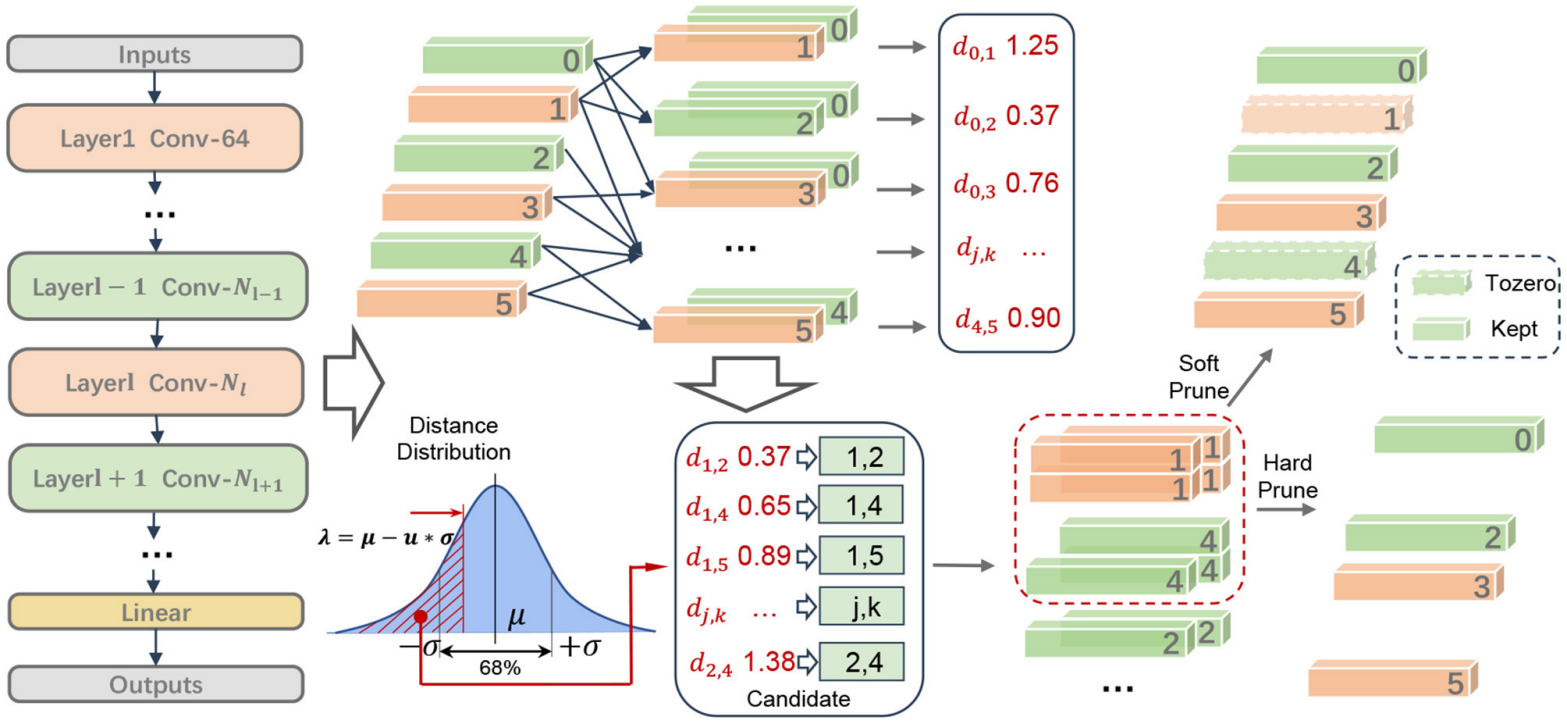
The pruning diagram of a convolutional layer. First, we compare all the filters in pairs, and then we count the distance set obtained, and put the filters corresponding to the distance value below the soft threshold into the set to be pruned. Finally, the filters with a higher frequency are considered redundant. The two methods of soft pruning and hard pruning are used to deal with redundant filters.

This paper focuses on the above problems and aims to improve the network performance under the same compression ratio. Therefore, we propose a channel pruning framework based on filter similarity, and optimize the pruning redundancy criterion, pruning strategy, pruning structure and pruning process, as shown in Figure 1. Specifically, in the pruning criteria, different from previous works which used precise rules to sort filters, we consider the problem from another perspective, focusing on the correlation of filters in one layer, and propose that two filters with high similarity extract similar features, and the extracted features can replace each other. In the pruning strategy, we do not need to specify the pruning rate of each layer, and automatically determine the pruning rate of each layer after determining the filter to be deleted according to the redundancy condition. In the pruning structure, we propose fine-grained pruning for special structures, in which the input and output channels of each block are calculated according to the redundancy condition constraints and then pruned in units of groups, thus increasing the reliability selection space for pruning channels. In addition, in the pruning process, for the situation that a lot of fine tuning is needed in the existing works, we perform a small amount of fine-tuning after each pruning of the whole network, which improves the efficiency of pruning. To summarize, our main contributions are as follows:

- We propose a novel method for estimating filter redundancy based on filter similarity, which does not rely on precise criteria to evaluate the importance of filters.
- The algorithm adaptively obtains the pruning rate of the layers according to the redundancy degree of each layer, which is difficult to determine in previous methods.
- The algorithm optimizes the channel pruning strategy of the special network structure, allowing the input and output channels of the residual block to be removed, further increasing the pruning space.
- The algorithm prunes the filters of the entire network at one time, and adopts two different pruning processes, hard pruning and soft pruning, which greatly reduces the large amount of fine-tuning caused by layer-by-layer pruning.

## 2. Related work

The typical work of network pruning is weight pruning and filter pruning. Weight pruning prunes individual parameter in the network to obtain a sparse weight matrix. Different from weight pruning, filter pruning removes the entire filter according to a certain measure. Filter pruning significantly reduces storage usage and decreases the computational cost of online inference. The key to filter pruning is the selection of filters, which should yield the highest compression ratio with the lowest compromise in accuracy. Based on the design of the filter importance criterion, we empirically divide the filter pruning into the following categories.

### 2.1. Based on magnitude

The simplest heuristic is to evaluate importance according to the absolute value of the parameter (or feature output) and then prune the part below the threshold by the greedy method, which is called amplitude-based weight pruning. Li et al. (2016) proposed using the absolute value of the weight as a measure of its importance (Zhang H. et al., 2018; Zhang et al., 2022). For structured pruning, group LASSO is often used to obtain structured sparse weights, such as in Liu et al. (2015) and Wen et al. (2016). Liu et al. (2017) introduced a channel scaling factor in the BN layer and pruned the corresponding weights with small scaling factors. In addition, the importance evaluation can also focus on the activation value. Hu et al. (2016) proposed using the average percentage of zero value (APoZ) to measure the importance of the activation value.

### 2.2. Based on loss function

The assumption based on absolute value judgment is that the smaller the absolute value of a parameter is, the smaller the influence on the final result. We call this the “smaller-norm/less-important” criterion, but this assumption is not necessarily true (as discussed in Ye et al., 2018). Another method is to consider the impact of parameter pruning on loss. LeCun et al. (1990) and Hassibi and Stork (1993) proposed the OBD and OBS methods, respectively, which measure the importance of weights in a network based on the second derivative of the loss function relative to the weight (the Hessian matrix for the weight vector). The method of Molchanov et al. (2016) was also based on Taylor expansion, but it utilized the absolute value of the first-order term in the expansion of the objective function relative to the activation function as the criterion for pruning. This avoids the calculation of second-order terms (i.e., the Hessian matrix). Lee et al. (2018) regarded the absolute value of the derivative of the normalized objective function with respect to the parameter as a measure of importance.

### 2.3. Based on the reconstructability of the feature output

The third method is to consider the impact on the rebuildability of the feature output, that is, minimizing the reconstruction error of the pruned network for the feature output. Typically, methods such as those of Luo et al. (2017) and He et al. (2017) identify channels that need to be pruned by minimizing feature reconstruction errors. Yu et al. (2018) proposed the NISP algorithm by minimizing the reconstruction error of the penultimate layer of the network, and back-propagating the importance information to the front to determine the channel to be pruned. Zhuang et al. (2018) proposed the DCP method. On the one hand, additional discriminative perception loss is added to the middle layer (to strengthen the discriminative ability of the middle layer), and on the other hand, the loss function of the error is also considered. The gradient information of the two losses is synthesized for the parameters, and the channels that need to be pruned are determined.

### 2.4. Other criteria

There are also other criteria based on the weights of the importance of ranking. He et al. (2019) proposed a filter pruning via geometric median (FPGM) method, the basic idea of which was to remove redundant parameters based on the geometric median. Lin et al. (2020) developed a method that was mathematically formulated to prune filters with low-rank feature maps. Wang et al. (2021) statistically modeled the network pruning problem in a redundancy reduction perspective and finded that pruning in the layer with the most structural redundancy outperforms pruning the least important filters across all layers. Cai et al. (2022) utilized a variant of the pruning mask as a prior gradient mask to guide fine-tuning. The disadvantage of the greedy algorithm is that it can only find local optimal solutions and ignores the relationship between the parameters. Some studies have aimed to consider the interrelationships among parameters to find a better global solution. Peng et al. (2019) proposed the CCP method, which considers the dependence between channels and formalizes the channel selection problem as a constrained quadratic programming problem. Wang et al. (2018) and Zhuo et al. (2018) used spectral clustering and subspace clustering to explore the relevant information in the channels and feature maps, respectively. With the development of AutoML research, such as AMC (He et al., 2018b), RNP (Lin et al., 2017), and N2N learning (Ashok et al., 2017), these tasks are all attempts to automate part of the pruning process.

## 3. Methodology

In this section, we introduce in detail the pruning algorithm based on the similarity of filters. The algorithm uses the similarity between the convolution filters in the convolutional layer to obtain network compression recommendations.

### 3.1. Motivation

Unlike current views of parameter importance-based pruning, we show that the removal of any one of the channels will not significantly impair the representational power of the network as long as there are two sufficiently similar channels. We derive theoretical support to justify the reasonability of our similarity-based pruning approach. Assuming that the neural network has *L* convolutional layers, *N*_*l*_ and *N*_*l*+1_ represent the number of input channels and output channels of the *l*_*th*_ layer convolution layer, respectively. *F*^(*l, i*)^ represents the *i*_*th*_ filter of the *l*_*th*_ layer, and the corresponding input feature map can be expressed as $F^{\left(l,i\right)}\in {\mathbb{R}}^{H\times W\times B}$, where *H, W, B* represent the height and width of the feature maps, and the batch size, respectively. The tensor of the connections of the *l*_*th*_ and *l*+1_*th*_ layers can be parameterized by $W\in {\mathbb{R}}^{N_{l}\times N_{l+1}\times K\times K},1\leq l\leq L$.

Considering two consecutive convolutional layers and using non-linear activation *h*(•) after each linear convolution, then:


(2) $$\begin{array}{l} F^{\left(l+1,n_{l+1}\right)}=\underset{n_{l}\in \left\{1,\ldots ,N_{l}\right\}}{\sum }h\left(F^{\left(l,n_{l}\right)}\right)*W^{\left(n_{l},n_{l+1}\right)} \end{array}$$

where $W^{\left(n_{l},n_{l+1}\right)}\in {\mathbb{R}}^{K\times K}$ is the *n*_*l*_-dimensional weight of the *n*_*l*+1_-th convolution kernel, corresponding to the *n*_*l*+1_-th input feature map. We explore and analyze the loss of representational power brought about by removing one of two similar feature channels and its filter. Suppose that $F^{\left(l,i\right)}$ and $F^{\left(l,j\right)}$ are two similar channels, deleting the $F^{\left(l,i\right)}$, then for the pruned $F_{p}^{\left(l+1,n_{l+1}\right)}$ we have:


(3) $$\begin{array}{l} \begin{array}{c} \begin{array}{c} F_{p}^{\left(l+1,n_{l+1}\right)}=h\left(F^{\left(l,j\right)}\right)*\left(W^{\left(i,n_{l+1}\right)}+W^{\left(j,n_{l+1}\right)}\right)\\ \;+\underset{n_{l}\neq i,j}{\sum }h\left(F^{\left(l,n_{l}\right)}\right)*W^{\left(n_{l},n_{l+1}\right)} \end{array} \end{array} \end{array}$$

We use mean squared error (MSE) to quantify the loss of the two feature maps before and after pruning:


(4) $$\begin{array}{l} L\left(F^{\left(l+1,n_{l+1}\right)},F_{p}^{\left(l+1,n_{l+1}\right)}\right)\\ ={\left(H_{l+1}\times W_{l+1}\times B\right)}^{-1}\times \|F^{\left(l+1,n_{l+1}\right)}-F_{p}^{\left(l+1,n_{l+1}\right)}{\|}_{2}^{2}\\ =\frac{1}{a_{l+1}}\|\left(h\left(F^{\left(l,i\right)}\right)-h\left(F^{\left(l,j\right)}\right)\right)*W^{\left(i,n_{l+1}\right)}{\|}_{2}^{2} \end{array}$$

where *a*_*l*+1_ = *H*_*l*+1_×*W*_*l*+1_×*B*. For each feature map $F_{p}^{\left(l+1,n_{l+1}\right)}$ in the *l*+1-th convolutional layer, the loss caused by removing the feature map $F^{\left(l,i\right)}$ from the *l*-th convolutional layer, as defined in Equation (4), admits the following upper bound:


(5) $$\begin{array}{l} L\left(F^{\left(l+1,n_{l+1}\right)},F_{p}^{\left(l+1,n_{l+1}\right)}\right)\leq \varepsilon \times \underset{j\in \left\{1,\ldots ,N_{l}\right\}}{\min}L\left(F^{\left(l,i\right)},F^{\left(l,j\right)}\right) \end{array}$$

where $\varepsilon =\frac{a_{l}}{a_{l+1}}K^{2}||W^{\left(i,n_{l+1}\right)}|{|}_{2}^{2}$ and *K*^2^ corresponds to the size of each filter $W^{\left(n_{l},n_{l+1}\right)}$. Detailed derivation can be found in Appendix. We can conclude from Equation (5) that E is determined by the size of the feature maps, the *L*2-norm of the convolution kernel and its weights. In experiments, E is usually a value of the order of 10^−2^, which means that the loss of removing one of the similar channels is negligible, as long as there are sufficiently similar channels to replace it.

In practice, our goal is to find similar channels and remove one of them. However, computing the similarity of channels directly has two apparent limitations. First, the activations of feature maps are affected differently by different batches of data. Second, calculating the similarity between all channels is inefficient for current large CNN architectures. To solve these issues, we use the convolution kernel as a unit for comparison. It can be seen from Equation (2) that when the input feature maps are the same, the feature maps obtained by similar convolution kernels are also identical, and the parameters of the kernels are not affected by the data batch. Intuitively, we quantify the similarity of two kernels by Euclidean distance, which is more commonly used in the analyses that need to reflect a difference in dimensions. In addition, Euclidean distance measures the distance between points in multidimensional space and can remember the absolute difference of characteristics. Therefore, for the *l*_*th*_ convolutional layer:


(6) $$\begin{array}{l} D^{\left(l\right)}=dist\left(F_{l,j},F_{l,k}\right),0\leq j\leq N_{l+1},j\leq k\leq N_{l+1}\\ \;=\left\{\begin{array}{cccc} d_{0,1} & d_{0,2} & \cdots & d_{0,N_{it1}-1}\\ \; & d_{1,2} & \cdots & d_{1,N_{it+1}-1}\\ \; & \; & \; & \vdots \\ \; & \; & \ddots & d_{j,k}\\ \; & \; & \; & \vdots \\ \; & \; & \; & d_{N_{i+1}-2,N_{i+1}-1} \end{array}\right\} \end{array}$$

where


(7) $$\begin{array}{l} d_{j,k}=\sqrt{\overset{N_{l}}{\underset{n=1}{\sum }}\overset{K}{\underset{k_{1}}{\sum }}\overset{K}{\underset{k_{2}=1}{\sum }}|W^{j}\left(n,k_{1},k_{2}\right)-W^{k}\left(n,k_{1},k_{2}\right){|}^{2}} \end{array}$$

$W^{j}\left(n,k_{1},k_{2}\right)$ is each weight in the filter *F*^(*l, j*)^. For the *l*_*th*_ convolution layer, we obtain a set of distances *D*^(*l*)^, which contains the distances between the *j*_*th*_ filter and all other filters. The smaller the distance is, the more significant the similarity between the two filters, indicating that the filter has extracted features similar to those of other filters.

**Figure 2:**
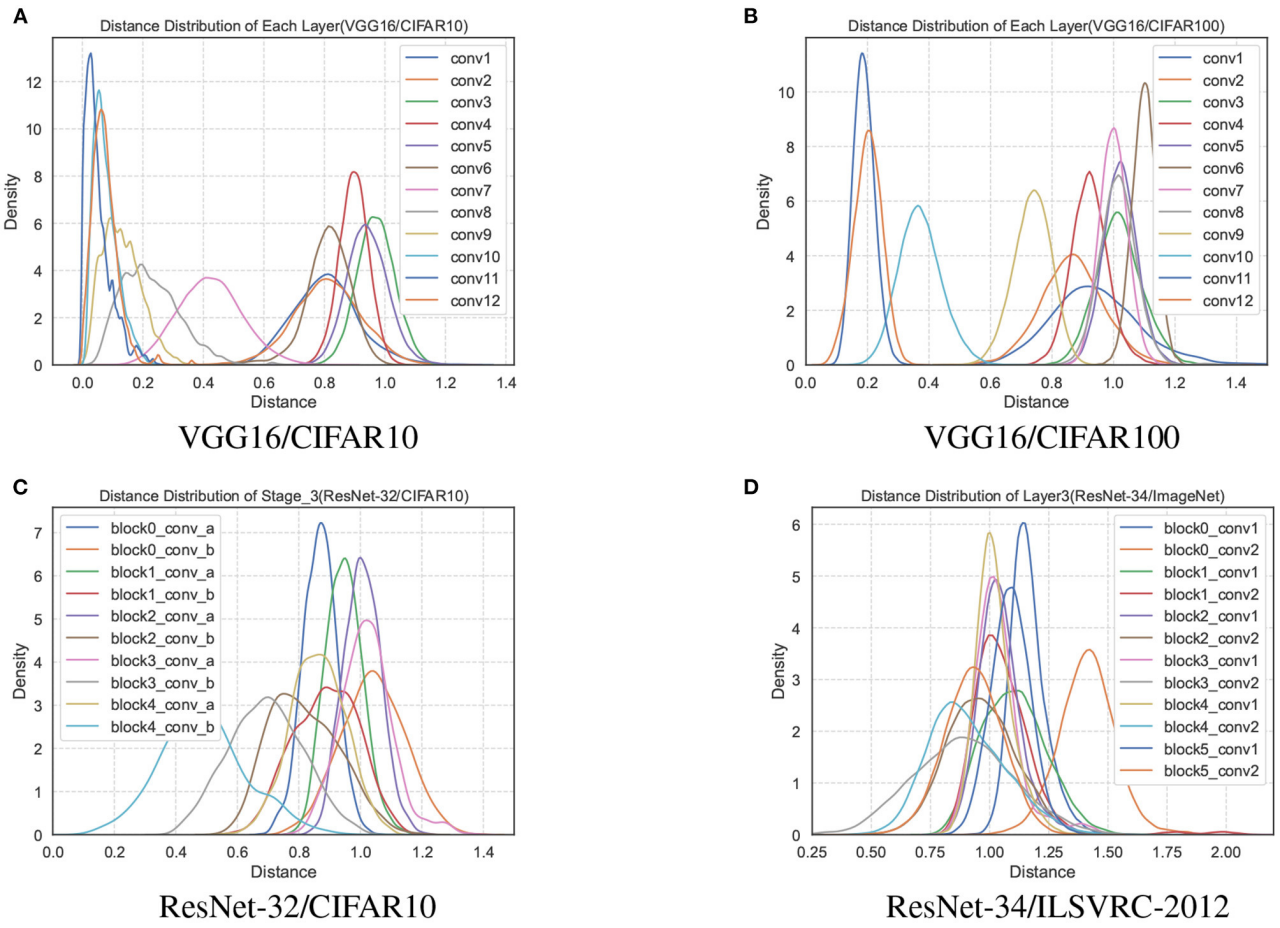
The distance distributions of each layer of the network model parameters trained by VGG16 on the CIFAR10 and CIFAR100 datasets are shown in **(A, B)**. **(C)** The distance distribution of all convolutional layers in the third stage of ResNet-32/CIFAR10. **(D)** The third layer of ResNet-34/ILSVRC-2012.

We remove the repeated distance with the same subscript in *D*^(*l*)^, and perform statistical analysis on all values in the set. Statistics show an interesting phenomenon that the distance distribution of each layer is an approximately Gaussian distribution in the trained network, as shown in Figure 2. The distance sets *D*^(*l*)^ of different layers in the network are distributed differently, and the mean value even differs by an order of magnitude. However, the distance distribution between the filters has partial jitters since the convolutional layers, such as conv1 and conv2 of the VGG16, are affected by the input data.

### 3.2. Filter pruning based on similarity

After the distance distribution of each convolutional layer is obtained, how great can the distance between the two filters be determined to be similar? One of the methods is to get a minimum distance value *min*[*D*^(*l*)^] each time, that is, to remove one filter each time until the set requirement is reached. That is inefficient and laborious for network structures with thousands of convolution kernels. To obtain a set of redundant filters simultaneously, we first need to set a threshold λ, and a pair of filters corresponding to a distance less than this threshold are judged to be similar. Since the distance distribution of each convolutional layer is different, simply specifying the threshold of each layer will bring more hyperparameter problems. How can a reasonable threshold be set for each layer more efficiently with fewer hyperparameters?

Inspired by the empirical rule (3σ) of a Gaussian distribution, the probability of falling within [μ−σ, μ+σ] is 0.68:


(8) $$\begin{array}{l} P^{\left(i\right)}\left(\mu -\sigma \leq x\leq \mu +\sigma \right)=0.68,x\in D^{\left(l\right)} \end{array}$$

we set a scaling factor α such that λ = μ−ασ∈(−∞, μ], and then α ∈ [0, +∞),


(9) $$\begin{array}{l} P^{\left(l\right)}\left(d_{j,k}^{\left(l\right)}\leq \lambda \right)=\frac{1}{\sqrt{2\pi }\sigma }\int_{-\infty }^{\lambda }\exp\left(-\frac{{\left(x-\mu \right)}^{2}}{2\sigma^{2}}\right)dx\\ \;\underline{\underline{t=\frac{x-\mu }{\sigma }}}\frac{1}{\sqrt{2\pi }}\int_{-\infty }^{\frac{\lambda -\mu }{\sigma }}\exp\left(-\frac{t^{2}}{2}\right)dt\\ \;=\frac{1}{\sqrt{2\pi }}\int_{-\infty }^{-\alpha }\exp\left(-\frac{t^{2}}{2}\right)dt\\ \;=\Phi (-\alpha )=1-\Phi (\alpha ) \end{array}$$

where Φ(•) is the distribution function of the standard normal distribution, it can be obtained from checking the Standard normal distribution table: when α = 0, λ = μ, *P* = 0.5; and α → +∞, λ → −∞, *P* → 0. If $d_{j,k}^{\left(l\right)}<\mu -\alpha^{*}\sigma$, the filters corresponding to $d_{j,k}^{\left(l\right)}$ in the shaded part of Figure 1 are judged to be similar, and then $d_{j,k}^{\left(l\right)}$ is selected as the candidate set $D_{select}^{\left(l\right)}$:


(10) $$\begin{array}{l} \begin{array}{c} d_{j,k}^{\left(l\right)}\in D_{select}^{\left(l\right)}\\ j,k\in F_{select}^{\left(l\right)} \end{array} \end{array}$$

$F_{select}^{\left(l\right)}$ is the set of indexes of the corresponding filters in $D_{select}^{\left(l\right)}$. We use a hyperparameter α to get equal-probability candidate sets in different layers for different distance distributions in each layer.

It can be seen in the experiment that a filter satisfies similar conditions simultaneously with multiple filters, but how can we determine the final deleted filters in the candidate set. For the *l*_*th*_ layer, we count the number of times of the *j*_*th*_ appears in $F_{select}^{\left(l\right)}$, denoted by $C_{j}^{\left(l\right)}$. Under extreme circumstances, if $d_{j,k}^{\left(l\right)}<\lambda \left(0\leq k\leq N_{l+1}-1,k\neq j\right)$ holds for the distance between the *j*_*th*_ filter and all other filters, then $C_{j}^{\left(l\right)}=N_{l+1}-1$. We use the proportional factor *r* ∈ [0, 1] to represent the frequency of the *j*_*th*_ filter,


(11) $$\begin{array}{l} r=\frac{C_{j}^{\left(l\right)}}{N_{l+1}-1} \end{array}$$

If $C_{j}^{\left(l\right)}>r^{*}\left(N_{l+1}-1\right)$, then $j\in F_{pruned}^{\left(l\right)}$, $F_{pruned}^{\left(l\right)}$ is the set of final pruning filters. The above algorithm obtains a set of redundant filters for one convolution layer in the network structure, and the schematic diagram of the pruning process of each layer is shown in Figure 1.

### 3.3. Compression recipes

In addition to the judgment method of network parameter redundancy, the pruning strategy, implementation and network structure are also essential factors that affect the compression performance. As the pruning rate increases, network performance loss increases, and the redundant judgment of parameters is also prone to deviation when the network parameters deviate from the optimal point. Previous work uses layer-by-layer pruning and fine-tuning strategies or retraining to reduce the judgment error caused by performance loss and iterates this process until the target compression rate is achieved. However, when the iteration parameter setting is small and the target compression rate is significant, the pruning period will greatly increase, and the training time cost will be very high. Therefore, we prune all layers at once instead of layer-by-layer pruning and fine-tuning, significantly reducing the pruning cost. After complete pruning, the computation and parameter quantity of the whole network are calculated. If the set pruning requirements are met, the pruning is completed; otherwise, the redundant filters will continue to be searched for further pruning on the network structure of the last pruning until the set pruning requirements are met (computational cost reduction or parameter reduction), as shown in Algorithm 1.

**Algorithm 1:**
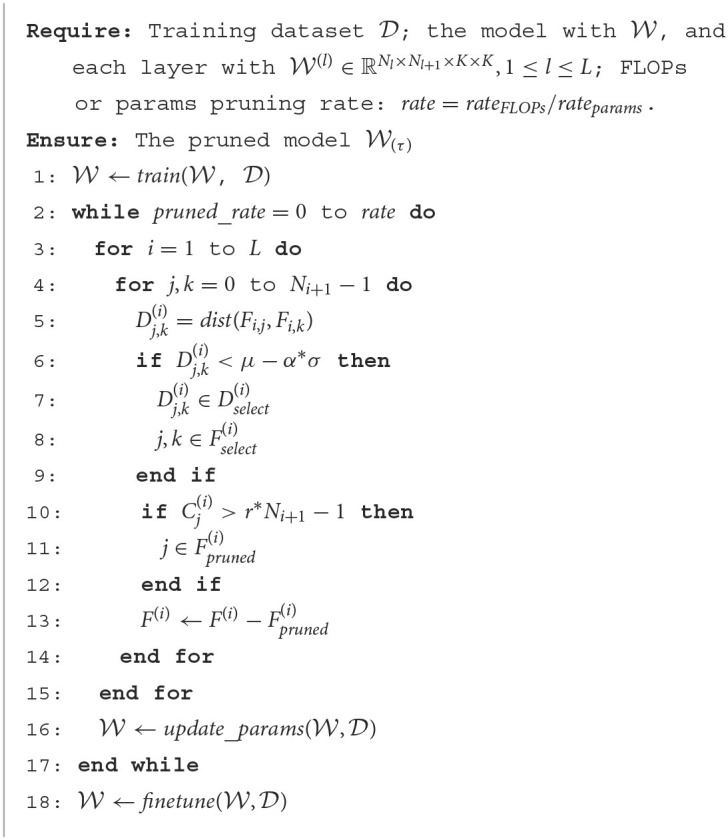
Iterative pruning algorithm.

In the implementation of pruning, He et al. (2018a) proposed not to directly delete the pruned parameters in the pruning process, which increases the fault tolerance of judgment. Many current works are based on soft pruning implementations, and for a fair comparison, we propose an iteration pruning strategy based on soft pruning. In the experiment, it is found that although the filters set to zero in the previous iteration are not deleted, they will not change in the subsequent fine-tuning no matter how the network is updated, which affects the distance calculation and redundant judgment. To solve this problem, we set a mask for each filter, the pruned filters are 0, and the others are 1, and the mask is updated by the algorithm in real-time in each iteration. When calculating the distance between the filters in one layer, the distance will be multiplied by the mask value corresponding to the two filters at the same time,


(12) $$\begin{array}{l} \begin{array}{l} d_{j,k}^{\left(l\right)}=dist\left(F_{l,j},F_{l,k}\right)*mask_{j}*mask_{k}\\ =\{\begin{array}{l} 0,\;mask_{j}=0\;or\;mask_{k}=0\\ dist\left(F_{l,j},F_{l,k}\right),mask_{j}\neq 0\;and\;mask_{k}\neq 0 \end{array} \end{array} \end{array}$$

In the distance set *D*^(*l*)^, the distance $d_{j,k}^{\left(l\right)}$ between a filter with a mask of zero and any other filter is zero. Before the next step of obtaining the distance statistics, the algorithm ignores a value of zero for $d_{j,k}^{\left(l\right)}$, which is equivalent to allowing only the unpruned filters to participate in the subsequent pruning.

In pruning structure, some networks with special structures, such as ResNet and DenseNet, improve the efficiency and performance, but also make pruning more challenging. Only pruning the middle layer in the block is currently the most used strategy, but the filters between blocks are not easily pruned due to excessive constraints. We propose a more flexible pruning strategy, which is pruned in units of blocks, increasing the selection space of pruned filters under the guarantee rules. First, we calculate the pruning rate of the middle layers of all blocks in a group according to the filter redundancy determination algorithm proposed in the previous section, and then take the minimum value as the group's pruning rate *rate*_*group*_. And then, the number of filters $card\left(F_{pruned}^{\left(l\right)}\right)$ to be pruned at any *l*_*th*_ layer in the group can be obtained:


(13) $$\begin{array}{l} card\left(F_{pruned}^{\left(l\right)}\right)=rate_{group}*N_{l+1} \end{array}$$

**Figure 3:**
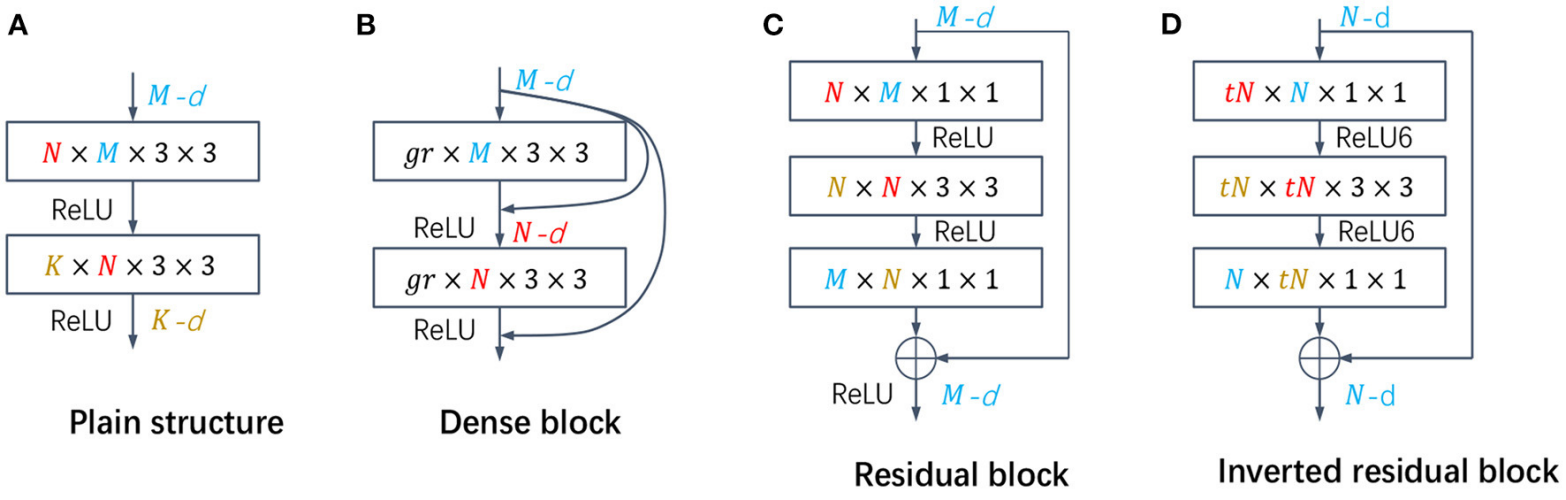
**(A)** The plain structure in VGG, and the number of output channel pruning in each layer is directly calculated by the algorithm. **(B)** The dense block, the number of output channels of each layer is fixed (*gr*), and the number of input channels is calculated by the algorithm. **(C, D)** Residual block and inverted residual block, respectively. The pruning rate of all layers in the block is the same, and the number of input channels and output channels of each block is guaranteed to be the same.

For the *l*_*th*_ layer, $F_{selected}^{\left(l\right)}$ can be obtained by Equation (10), and the number of occurrences $C_{j}^{\left(l\right)}$ of the *j*_*th*_ filter in $F_{selected}^{\left(l\right)}$ can be sorted. The final pruned filters $F_{pruned}^{\left(l\right)}$ intercept the top $card\left(F_{pruned}^{\left(l\right)}\right)$ filters from $F_{selected}^{\left(l\right)}$. The specific pruning mode of the different structures is shown in Figure 3.

The algorithm calculates the redundant filters of the whole network at one time instead of layer-by-layer, and then prunes or sets them to zero. The FLOPs and parameters reduction for the entire network is calculated after one iteration. If the set pruning rate is reached, the pruning is completed; otherwise, the parameters are updated to find more similar filters for further pruning. Then pruning is performed again until the set pruning rate is reached. After all pruning is completed, only a small amount of fine-tuning is required, as shown in Algorithm 1. In addition, we compare the current works with our proposed method from the aspects of criteria, whether to manually set the pruning rate of each layer, whether to process the residual structure, and the pruning method, as shown in Table 1. The proposed method optimizes and improves the pruning criterion, pruning rate setting, special structure processing, and pruning method.

**Table 1:** Comparison of our proposed method with current works.

**Method**	**Criterion**	**Manually specify the pruning rate?**	**Residual structure processing?**	**Pruning method**
L1	L1-norm	Yes	No	Hrad
Taylor	Taylor expansion	Yes	No	Hrad
ThiNet	Reconstruction error	Yes	No	Hrad
SFP	L2-norm	Yes	No	Soft
FPGM	Geometric median	Yes	No	Soft
HRank	Feature maps' average rank	Yes	No	Hrad
SRR-GR	L1-norm	No	No	Hrad
PGMPF	L2-norm	Yes	No	Soft
Ours	Filter similarity	No	Yes	Hrad/Soft

## 4. Experiments

We evaluate the effectiveness of our algorithm on CIFAR-10 (Krizhevsky et al., 2009), CIFAR-100 (Krizhevsky et al., 2009), and ILSVRC-2012 (Russakovsky et al., 2015) datasets using representative CNN architectures: VGGNet (Simonyan and Zisserman, 2014), ResNet (He et al., 2016), and DenseNet. CIFAR10 contains 50,000 training images and 10,000 testing images of size 32 × 32, which are categorized into 10 different classes. CIFAR100 is similar to CIFAR-10 but has 100 classes. ImageNet contains 1.28 million training images and 50 k validation images of 1,000 classes. VGGNet and ResNet represent two typical network structures with single branch and multiple branches respectively, and DenseNet prunes the input channels.

We calculate the size and computational complexity of the network through the number of network parameters and floating point operations (FLOPs) for one forward propagation. For the *l*_*th*_ convolutional layer,


(14) $$\begin{array}{l} \begin{array}{ll} FLOPs & =HW\left(C_{in}K^{2}+1\right)C_{out}\\ params & =\left(C_{in}K^{2}+1\right)C_{out} \end{array} \end{array}$$

*H* and *W* are the length and width of the input feature map, respectively, and *C*_*in*_, *C*_*out*_ are the number of input and output channels of the *l*_*th*_ convolutional layer, which correspond to the number of filters *N*_*l*_ and *N*_*l*+1_.

We evaluate the performance of the convolution kernel pruning method by using the method of parameter quantity or the drop rate of computation, and different performance indicators can be used according to the requirements of different scenarios:


(15) $$\begin{array}{l} \begin{array}{ll} rate_{FLOPs} & =1-\frac{FLOPs_{original}}{FLOPs_{compressed}}\\ rate_{params} & =1-\frac{params_{original}}{params_{compressed}} \end{array} \end{array}$$

Different pruning methods use pre-trained models or self-trained models as the baseline network. Due to the different training parameters (e.g., different learning rates, training times, data augmentations, etc.) and different experimental frameworks (TensorFlow, PyTorch, etc.), the Top-1 and Top-5 accuracies of the baseline network reported in the original papers are different. To make a fair comparison, we evaluate the effectiveness of pruning using the drop rate of Top-1 and Top-5 accuracy on the test set, which is the accuracy difference between the baseline network and the compressed network. Under the same compression rate, the smaller the difference, the better the pruning effect. All the comparison results in this paper are directly quoted from the original paper of the related method or the official code reproduction. All experiments are implemented on four NVIDIA TITAN Xp GPUs using PyTorch.

### 4.1. Results on the CIFAR-10/100 datasets

We analyze the performance on the CIFAR datasets with VGG16, DenseNet-40, and ResNet-32/56/110. All the networks are trained using SGD with Nesterov momentum (Sutskever et al., 2013) 0.9, a weight decay parameter of 10^−4^, and an initial learning rate of 0.1. The learning rate is set to 0.001 when updating parameters or fine-tuning. For VGG16 and DenseNet-40, the baseline network is trained for 300 epochs with a batch size of 256. And for ResNet, the baseline network is trained for 200 epochs with a batch size of 256.

#### 4.1.1. CIFAR10

We make a comparison with methods using hard pruning strategies, such as L1 (Li et al., 2016), the method of Molchanov et al. (2016), and with some current soft pruning methods, such as SFP (He et al., 2018a), FPGM (He et al., 2019), and HRank (Lin et al., 2020), and SRR-GR (Wang et al., 2021). In the VGG16/DenseNet experiment, α is set to 1, *r* is set to 0.35. And in ResNet, α is set to 1, *r* is set to 0.3. We adopt *rate*_*FLOPs*_ as constraints and report *rate*_*params*_ at the same time.

Results on CIFAR10 dataset are shown in Table 2. It can be observed that our proposed algorithm outperforms other methods under different networks and with similar or even higher compression ratios. In VGG16 with a plain structure, the performance of the similarity-based redundancy determination method far exceeds the other pre-defined determination methods, which indicates that the similarity-based determination method can effectively identify redundant parameters. On pruning strategy, soft pruning and hard pruning have little difference in performance under the same FLOPs pruning rate constraint. For example, at a pruning rate of 42.5%, the pruning performance of soft pruning is even worse than hard pruning. Moreover, there is little difference in performance between the evaluation criteria at a low pruning rate, but as the pruning rate increases, the judgment criteria have a more significant impact on the pruning performance.

**Table 2:** Comparison of the results of different network structures on the CIFAR10 dataset.

**Model**	**Method**	**Prune**	**Top-1 (↓) (%)**	**FLOPs (↓) (%)**	**Params (↓) (%)**
VGG16	**L1 (Li et al., 2016)**	**✓**	**0.15**	**34.20**	**64.00**
	**Ours**	✓	**0.17**	**42.47**	43.95
	L1 (Li et al., 2016)	✓	3.66	83.51	83.46
	(Molchanov et al., 2016)	✓	2.78	78.03	84.56
	**Ours**	✓	**1.74**	**81.62**	**82.33**
	FPGM (He et al., 2019)	✗	0.34	34.20	64.0
	**Ours**	✗	**0.17**	**42.48**	43.96
	HRank (Lin et al., 2020)	✗	2.73	76.50	92.0
	**Ours**	✗	**1.56**	**79.68**	81.64
	**Ours**	✗	**1.93**	**88.99**	**92.70**
ResNet-32	L1 (Li et al., 2016)	✓	11.81	43.76	44.72
	**Ours**	✓	**0.31**	43.47	43.61
	SFP (He et al., 2018a)	✗	0.55	41.50	–
	FPGM (He et al., 2019)	✗	0.70	53.2	–
	**Ours**	✗	**−0.29**	50.36	**55.71**
ResNet-56	L1 (Li et al., 2016)	✓	1.75	27.60	–
	SFP (He et al., 2018a)	✗	1.33	52.60	–
	FPGM (He et al., 2019)	✗	0.66	52.60	–
	HRank (Lin et al., 2020)	✗	0.09	50.00	42.40
	SRR-GR (Wang et al., 2021)	✗	−0.37	53.8	–
	**Ours**	✗	**−0.64**	**61.10**	**58.31**
ResNet-110	L1 (Li et al., 2016)	✓	0.61	38.60	–
	**Ours**	✓	1.65	**60.70**	**60.80**
	SFP (He et al., 2018a)	✗	0.30	40.80	–
	FPGM (He et al., 2019)	✗	−0.05	52.30	–
	HRank (Lin et al., 2020)	✗	0.85	68.60	42.40
	**Ours**	✗	**0.53**	**71.69**	**76.06**
DenseNet-40	HRank (Lin et al., 2020)	✗	0.57	40.80	36.5
	**Ours**	✗	**0.37**	**45.24**	**41.04**
	HRank (Lin et al., 2020)	✗	1.13	61.00	53.80
	**Ours**	✗	**0.90**	**62.22**	**62.02**

The "✓" indicates hard-pruning and "✗" indicates soft-pruning. The "(↓)" denotes the drop between baseline and the pruned model. A negative value in "Top-1(↓)(%)" indicates an improve model accuracy over the baseline model. The "-" denotes results are not reported in original papers. Other tables follow the same convention. The bold values indicate that experimental results are better than other methods.

In ResNet, the processing of the pruning structure and the pruning strategy also have an impact on the compression performance in addition to the criterion. The performance of hard pruning for L1 and ours is slightly worse than that of the soft pruning strategy. SFP uses the pruning principle with a small absolute value and does not prune the channels between the residual blocks, thus the performance is the worst. FPGM and HRank employ more effective criteria and a lot of fine-tuning, and the performance is improved. We achieve superior compression performance over existing work using a similarity-based determination method and fewer fine-tuning epochs with the same soft-tuning implementation strategy. For DenseNet, where the input channels need to be pruned, we more effectively identify the redundant input channels while achieving excellent compression performance. Overall, soft pruning achieves higher pruning rates with similar accuracy than hard pruning. The criterion has a greater impact on the plain structure, in which the number of channels between layers is not constrained. The pruning performance of models with unique structures is affected by the judging criteria and the pruning strategy.

#### 4.1.2. CIFAR100

The results on the CIFAR100 dataset are shown in Table 3. Compared to the CIFAR10 dataset, CIFAR100 is more challenging for pruning due to more categories. We compare with L1 (Li et al., 2016), the method of Molchanov et al. (2016), SFP (He et al., 2018a), FPGM (He et al., 2019), and PGMPF (Cai et al., 2022) on VGG16 and ResNet32/56/110. In the VGG16 experiment, α is set to 1, *r* is set to 0.35, and in ResNet, α is set to 1, *r* is set to 0.3. All the data in the table are obtained under the same number of fine-tuning according to the public code. The parameters not given in the table are because the code or the paper does not give the specific calculation process. It can be observed that our method still outperforms other existing methods when reaching similar or higher pruning rates. Compared with the CIFAR10 dataset, the gap between different judgment criteria methods is more prominent, and even the accuracy gain brought by the increased number of fine-tuning still cannot compensate for the performance loss of the network due to inaccurate pruning. For example, SFP reduces the accuracy by 2.21% under half the FLOPs compression on ResNet32. FPGM still has an accuracy loss of 0.16% with the increased number of fine-tuning. However, the accuracy of our method has not decreased but increased, which can reflect the differences between different evaluation criteria. At the same time, the network is more sensitive to pruning on larger datasets, and the redundancy of the network does not increase with the depth of the network, which brings more difficulty to the judgment of parameter redundancy. For ResNet110, while the pruning rate is reduced compared to ResNet56, the network performance also drops significantly.

**Table 3:** Comparison of pruned ResNet on CIFAR100.

**Depth**	**Method**	**Prune**	**Top-1 (↓) (%)**	**Top-5 (↓) acc (%)**	**FLOPs (↓) (%)**	**Params (↓) (%)**
VGG16	L1 (Li et al., 2016)	✓	2.24	1.27	50.44	50.23
	(Molchanov et al., 2016)	✓	2.36	1.42	40.25	47.36
	**Ours**	✓	**1.69**	**1.72**	**51.99**	**68.79**
	FPGM (He et al., 2019)	✗	2.06	1.73	48.93	–
	PGMPF (Cai et al., 2022)	✗	0.35	–	48.20	–
	**Ours**	✗	**0.34**	**1.25**	**52.80**	**62.97**
ResNet-32	L1 (Li et al., 2016)	✓	18.37	11.47	43.76	44.16
	**Ours**	✓	**2.74**	**1.73**	43.45	43.38
	SFP (He et al., 2018a)	✗	2.21	1.12	53.16	–
	FPGM (He et al., 2019)	✗	0.16	-0.63	53.16	–
	**Ours**	✗	**−0.59**	−0.07	50.51	**53.25**
ResNet-56	SFP (He et al., 2018a)	✗	1.05	−0.16	63.16	–
	FPGM (He et al., 2019)	✗	1.33	−0.10	63.16	–
	PGMPF (Cai et al., 2022)	✗	2.71	–	52.6	–
	**Ours**	✗	**0.71**	1.03	**64.98**	**61.45**
ResNet-110	**Ours**	✗	0.98	0.65	59.23	56.70

The bold values indicate that experimental results are better than other methods.

### 4.2. Results on ILSVRC-2012

In the experiments, we use ResNet-18/34/50 to demonstrate the proposed pruning performance on a large-scale dataset, ILSVRC-2012 (Russakovsky et al., 2015). All the baseline networks are obtained by training 100 epochs with a batch size of 256. We follow the same parameter settings as [16] and [56], where the hyperparameter α is set to 1 and *r* is set to 0.35. We compare the proposed method with ThiNet (Luo et al., 2018), FPGM (He et al., 2019), MIL (Dong et al., 2017), PFEC (Li et al., 2016), CP (He et al., 2017), SFP (He et al., 2018a), HRank (Lin et al., 2020), PGMPF (Cai et al., 2022), and SRR-GR (Wang et al., 2021) and present the results in Table 4. All the results of the other methods in the table are directly from their reports in the literature. For ResNet with different depths, the hard pruning and soft pruning strategies are tested to make a fair comparison with other methods of different implementations. From the previous experiments on the CIFAR10/100 datasets, we conclude that the network performance is more sensitive to pruning in underfitted network structures. For ResNet18/34, our algorithm achieves the same FLOPs drop rate under the hard pruning strategy and achieves a smaller Top-1 accuracy drop rate than other methods using soft pruning strategies; in soft pruning, a better performance is still obtained with more pruned FLOPs than other methods. For ResNet50, the performance of the pruning algorithms is not very different, but our algorithm still achieves a better performance. For example, it reduces the computation by nearly half (53.05%), while the Top-1 accuracy loss is only 0.29%. Similarly, the final performance of the soft pruning strategy is still significantly better than that of the hard pruning strategy.

**Table 4:** Comparison of pruned ResNet on ILSVRC-2012.

**Model/ Data**	**Method**	**P.F**.	**Base top-1 acc(%)**	**Pruned top-1 acc(%)**	**Top-1 (↓)(%)**	**Base top-5 acc(%)**	**Pruned top-5 acc(%)**	**Top-5 (↓)(%)**	**FLOPs (↓)(%)**
ResNet18	MIL (Dong et al., 2017)	✓	69.98	66.33	3.65	86.94	89.24	2.30	34.6
	**Ours**	✓	**70.48**	**68.58**	**1.90**	**89.60**	**88.44**	**1.16**	**50.1**
	SFP (He et al., 2018a)	✗	70.28	67.10	3.18	89.63	87.78	1.85	41.8
	FPGM (He et al., 2019)	✗	70.28	67.81	2.47	89.63	88.11	1.52	41.8
	PGMPF (Cai et al., 2022)	✗	70.23	66.67	3.56	89.51	87.36	2.15	53.5
	**Ours**	✗	**70.48**	**68.96**	**1.52**	**89.60**	**88.55**	**1.05**	**52.85**
ResNet34	MIL (Dong et al., 2017)	✓	73.42	72.99	0.43	91.36	91.19	0.17	24.8
	PFEC (Li et al., 2016)	✓	73.23	72.17	1.06	-	-	-	24.2
	**Ours**	✓	**73.90**	**72.30**	**1.60**	**91.59**	**90.79**	**0.80**	**53.1**
	SFP (He et al., 2018a)	✗	73.92	71.83	2.09	91.62	90.33	1.29	41.1
	FPGM (He et al., 2019)	✗	73.92	72.11	1.81	91.62	90.69	0.93	41.1
	PGMPF (Cai et al., 2022)	✗	73.27	70.64	2.63	91.43	89.87	1.56	52.7
	**Ours**	✗	**73.90**	**72.80**	**1.10**	**91.59**	**91.04**	**0.55**	**52.07**
ResNet50	ThiNet (Luo et al., 2018)	✓	75.30	74.03	1.27	92.20	92.11	0.09	36.79
	CP (He et al., 2017)	✓	-	-	-	92.20	90.80	1.40	50.0
	**Ours**	✓	**75.82**	**74.74**	**1.08**	**92.95**	**92.28**	**0.67**	**40.78**
	SFP (He et al., 2018a)	✗	76.15	74.61	1.54	92.87	92.06	0.81	41.8
	FPGM (He et al., 2019)	✗	76.15	75.03	1.12	92.87	92.40	0.47	42.2
	HRank (Lin et al., 2020)	✗	76.15	74.98	1.17	92.87	92.33	0.54	43.76
	SRR-GR (Wang et al., 2021)	✗	76.13	75.76	0.37	92.86	92.60	0.19	44.10
	PGMPF (Cai et al., 2022)	✗	76.01	75.11	0.90	92.93	92.41	0.52	53.5
	**Ours**	✗	**75.82**	**75.53**	**0.29**	**92.95**	**92.83**	**0.12**	**53.05**

The bold values indicate that experimental results are better than other methods.

### 4.3. Ablation study

#### 4.3.1. Influence of hyperparameters

There are two hyperparameters α and *r* in the algorithm proposed in this paper. These two parameters together determine the pruning rate of each layer. From the introduction of the algorithm in Section 3, we only need to specify a set of α and *r* values for each network structure, to avoid manually specifying the pruning rate of each layer in the network. Next, we will discuss how to select the hyperparameters in the experiment and how their values affect the pruning rate. To explore the relationship more clearly, we choose to use the VGG16 to experiment on the CIFAR100 datasets.

**Figure 4:**
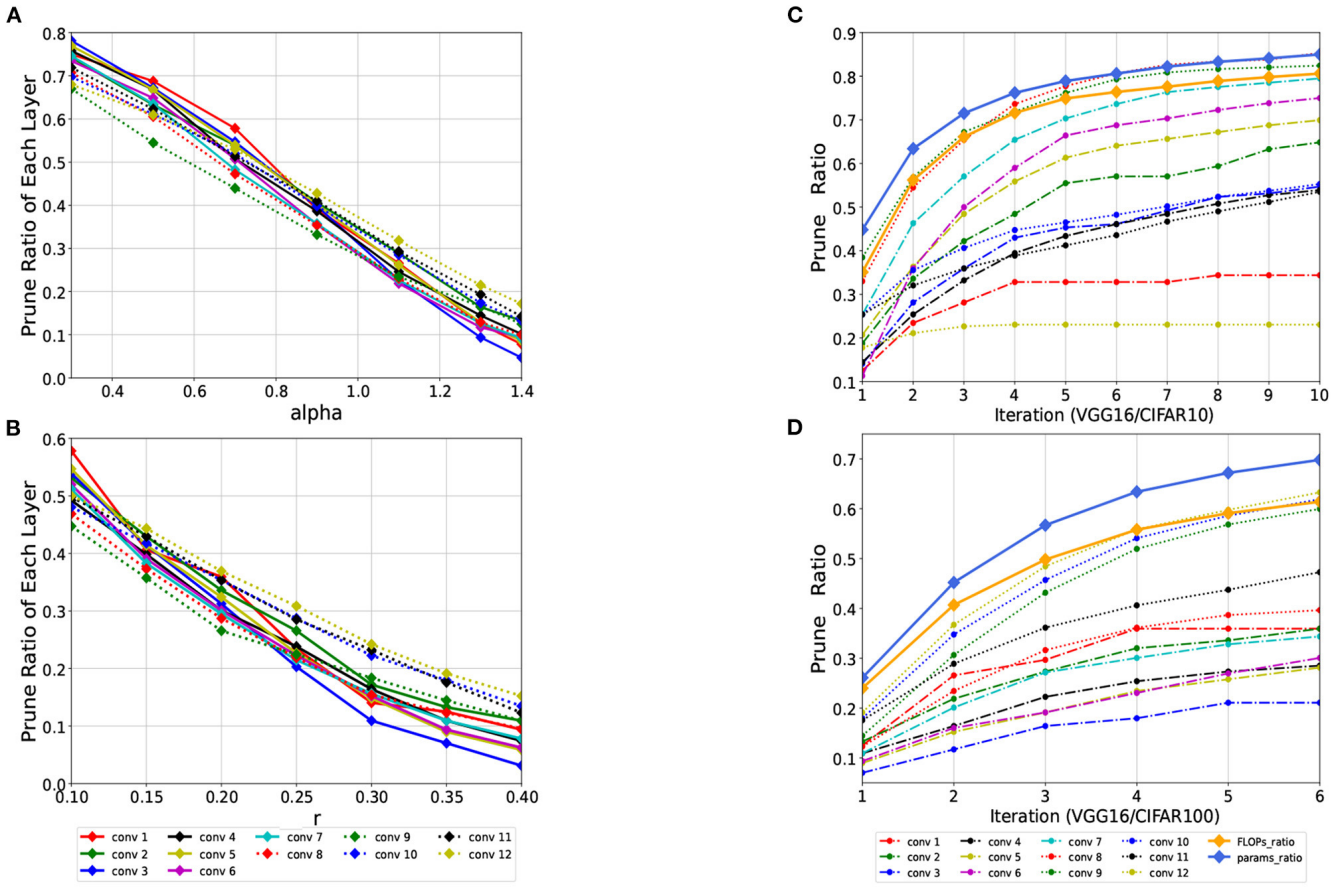
**(A)** The pruning rate of each convolutional layer with different values of α for the VGG16 on the CIFAR100 datasets at *r* = 0.35. **(B)** The pruning rate of different layers with different values of *r* when α = 1. **(C, D)** The pruning process of the VGG16 on the CIFAR10/100 datasets when *rate*_*FLOPs*_ is set to 0.8 and 0.6, respectively. On CIFAR10, the algorithm meets the pruning requirement after 10 iterations, while CIFAR100 exceeds 6 iterations. The pruning rate of each layer and the change process of *rate*_*FLOPs*_ and *rate*_*params*_ are also shown in the figure.

For different α values, the algorithm can obtain different candidate sets. This value determines how large the distance value of two filters should be if they will be selected to be pruned. The larger α is, the more filters are finally pruned. For a fixed value of *r*, the pruning rate of different layers obtained by different α is shown in Figure 4A. For different *r* values, different sets of final pruned channels can be obtained. For a convolution kernel, *r* determines how many other convolution kernels it is similar to, and it is regarded as a redundant convolution kernel. The larger *r* is, the fewer pruned filters are obtained. For a fixed α, the pruning rate of different layers obtained by different *r* values are shown in Figure 4B.

It can be inferred from the above figures that the values of *r* and α are correlated roughly linearly with the final pruning rate. These two hyperparameters together determine the pruning rate of each layer. According to the rules obtained from the experiments in the figure, we can take the appropriate *r* and α for different networks in later experiments. The algorithm does not need to precisely specify the exact values of *r* and α. Excellent experimental performance can be obtained when α is between 0.8 and 1.1 and *r* between 0.25 and 0.4, and the settings of α and *r* have a certain influence on the number of iterations. Once they are set, there is no need to specify the pruning rate of each layer, and the algorithm directly derives the filters that need to be pruned for each layer.

#### 4.3.2. Pruning rate change during iteration

The proposed algorithm determines the pruning rate of each layer adaptively without manual specification. After setting the FLOPs or parameters constraints, the algorithm automatically prunes the redundancies in each layer and calculates the FLOPs and parameters after each iteration. After several iterations, the set target is reached, and pruning is completed, thereby avoiding layer-by-layer pruning and much fine-tuning. As shown in Figure 4, pruning becomes increasingly difficult with increasing numbers of iterations, and the network performance becomes increasingly sensitive to pruning. In the last few iterations, only a small number of filters are pruned, which results in a significant decrease in accuracy. For different datasets, the redundancy of each convolutional layer for the same network structure is different. On the CIFAR10 datasets, the redundancy of the first few convolutional layers is higher, and the pruning rate is between 50 and 80%. However, the pruning rates of the first few layers on the CIFAR100 datasets are all below 40%.

#### 4.3.3. Feature map visualization and actual speedup

**Figure 5:**
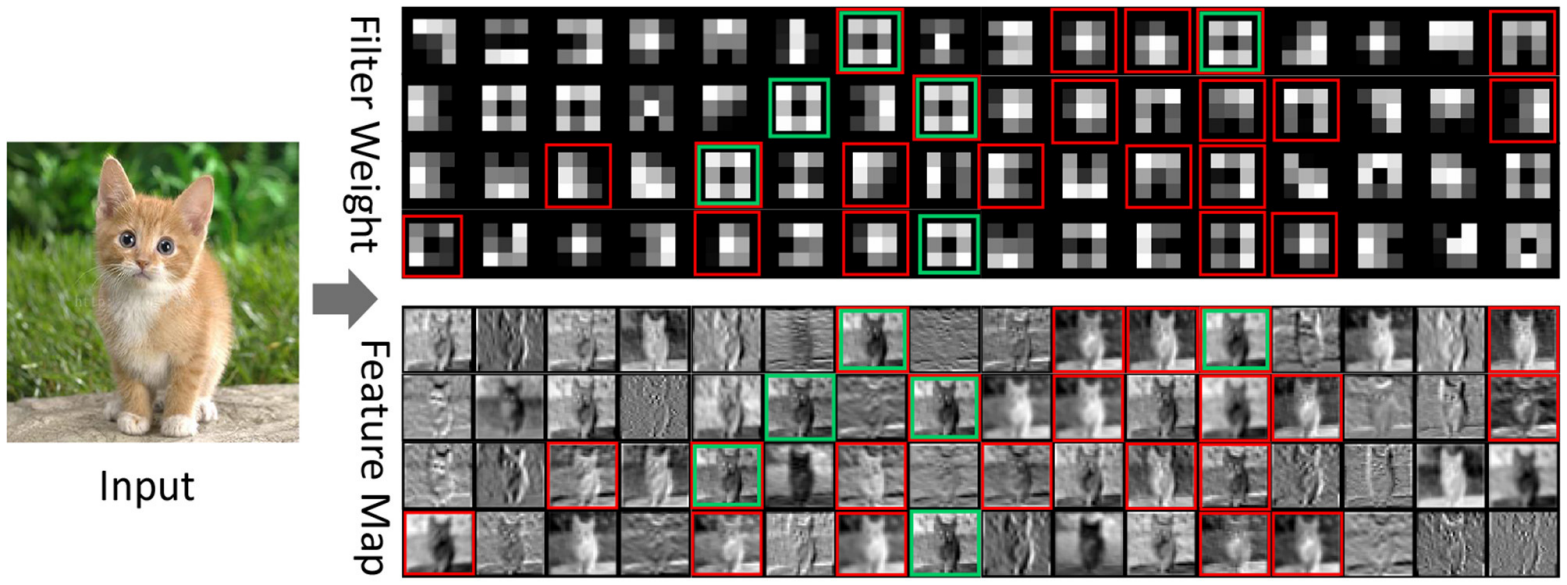
Visualization of the weights and feature maps of the first convolutional layer of the VGG16 on the CIFAR100 datasets. The first convolutional layer has 64 filters, and the filters with red bounding boxes are to be pruned. The green boxes are similar channels artificially determined according to the feature map and the convolution kernel, while the green boxes and red boxes are the channels identified and pruned by the algorithm.

To verify whether the filters identified by our proposed algorithm are truly redundant, we visualize the first layer of the convolution kernel and the corresponding feature map of the VGG16 on the CIFAR100 datasets. The part marked in red in the figure contains the pruned filters and the corresponding feature maps. We analyze the filters and the corresponding feature maps and find that there are multiple similar filters in the same convolution layer, and their corresponding feature maps are also quite similar. For example, comparing their weights and feature maps, the filters (7, 12, 22, 24, 37, 56) all extract the overall outline of the cat. Our algorithm prunes the filters (7, 12, 24, 37) and keeps the other two similar filters (22, 56), as shown in Figure 5.

We evaluate the actual speedup of our proposed method on the intelligent edge accelerator Jeston nano, as shown in Table 5. Since previous works used different GPUs and libraries, and pruned models are not readily available, we only report the inference time and speedup of the original model and the pruned model using our proposed method. It can be seen from the table that on edge devices, the inference speed of our proposed compression model is faster than that of the original model, but the actual speedup ratio cannot reach the theoretical reduction of calculation. The actual acceleration ratio of VGG is much smaller than the theoretical acceleration ratio, while the acceleration ratio of ResNet and DenseNet is comparable to the theoretical acceleration ratio. We believe that the gap between theoretical and actual speedup is mainly caused by the cache effect and memory accessing pattern in GPU, which is affected by the hardware itself, the network architecture, and Pytorch library implementation.

**Table 5:** Speedups of compressed network models on different datasets.

**Model/Dataset**		**Original time (ms)**	**Pruned time (ms)**	**Speedup**
CIFAR10	VGG16 (11.01%)	16.92	4.69	3.61 ×
	ResNet-32 (49.64%)	6.78	3.83	1.77 ×
	ResNet-56 (38.90%)	9.60	4.15	2.31 ×
	ResNet-110 (23.94%)	15.12	4.28	3.53 ×
	DenseNet-40 (37.98%)	23.26	9.78	2.38 ×
ImageNet	ResNet18 (47.15%)	45.15	23.39	1.93 ×
	ResNet34 (47.93%)	73.82	45.38	1.63 ×
	ResNet50 (46.95%)	165.31	97.61	1.69 ×

## 5. Conclusion

In this article, we propose a novel strategy for judging the redundancy of filters based on similarity. To obtain the redundant filters, we analyze the similarity distribution law for filters in a convolution layer, and obtain a compact network by pruning the redundant filters with certain strategies. A large number of experiments proved the effectiveness and flexibility of the method under the same experimental parameters and the performance does not depend on a large number of fine-tunings.

Although the pruning method we proposed does not need to specify the pruning rate of each layer, it still relies on the values of two hyperparameters. If different hyperparameters are specified for each layer according to the redundancy of each layer, the network will be further compressed. We plan to combine this method with reinforcement learning to automatically adjust the required parameters and improve the performance to a higher level. We performed simple statistical tests on similar filters to provide a basis for further pruning, which is far insufficient for complex CNNs. We will further analyze the filters' similarity data and combine the visual analysis of each layer to provide guidance for pruning.

## Data availability statement

The original contributions presented in the study are included in the article/supplementary material, further inquiries can be directed to the corresponding author/s.

## Author contributions

TW: writing—original draft. CS and PZ: writing—review and editing. All authors have read and agreed to the published version of the manuscript.

## Proof of the equation 5

For the *i*_*th*_ feature map ${ℱ}^{\left(l,i\right)}$ of the *l*_*th*_ layer, let $\delta_{i,j}^{\left(l\right)}=h\left(F^{\left(l,i\right)}\right)-h\left(F^{\left(l,j\right)}\right)$ and $\delta_{i,j}^{\left(l\right)}\left(p_{1},p_{2}\right)$ denote the image block centered at (*p*_1_, *p*_2_), then we have:

(16)
$$\begin{array}{l} \begin{array}{l} ℒ\left({ℱ}^{\left(l+1,n_{l+1}\right)},{ℱ}_{p}^{\left(l+1,n_{l+1}\right)}\right)\\ =\frac{1}{a_{l+1}}{\left\|\left(h\left({ℱ}^{\left(l,i\right)}\right)-h\left({ℱ}^{\left(l,j\right)}\right)\right)*W^{\left(i,n_{l+1}\right)}\right\|}_{2}^{2}\\ =\frac{1}{a_{l+1}}\sum_{\begin{array}{c} p_{1}\in \left\{1,\cdots ,H_{l+1}\right\}\\ p_{2}\in \left\{1,\cdots ,l_{l+1}\right\} \end{array}}{\left(\delta_{i,j}^{\left(l\right)}\left(p_{1},p_{2}\right)*W^{\left(i,n_{l+1}\right)}\right)}^{2}\\ =\frac{1}{a_{l+1}}\sum_{\begin{array}{c} p_{1}\in \left\{1,\cdots ,l_{l+1}\right\}\\ p_{2}\in \left\{1,\cdots ,l_{l+1}\right\} \end{array}}{\left|\langle \delta_{i,j}^{\left(l\right)}\left(p_{1},p_{2}\right),W^{\left(i,n_{l+1}\right)}\rangle \right|}^{2} \end{array} \end{array}$$

Applying Cauchy-Schwarz inequality, then:

(17)
$$\begin{array}{l} ℒ\left({ℱ}^{\left(l+1,n_{l+1}\right)},{ℱ}_{p}^{\left(l+1,n_{l+1}\right)}\right)\\ \leq \frac{1}{a_{l+1}}\sum_{\begin{array}{c} p_{1}\in \left\{1,\cdots ,H_{l+1}\right\}\\ p_{2}\in \left\{1,\cdots ,H_{l+1}\right\} \end{array}}{\left\|\delta_{i,j}^{\left(l\right)}\left(p_{1},p_{2}\right)\right\|}_{2}^{2}{\left\|W^{\left(i,n_{l+1}\right)}\right\|}_{2}^{2}k\\ =\frac{1}{a_{l+1}}{\left\|W^{\left(i,n_{l+1}\right)}\right\|}_{2}^{2}\sum_{\begin{array}{c} p_{1}\in \left\{1,\cdots ,H_{l+1}\right\}\\ p_{2}\in \left\{1,\cdots ,H_{l+1}\right\} \end{array}}{\left\|\delta_{i,j}^{\left(l\right)}\left(p_{1},p_{2}\right)\right\|}_{2}^{2} \end{array}$$

Actually $\delta_{i,j}^{\left(l\right)}$ appears at most *K*^2^ times in the convolution operation, except for the border. For activation functions commonly used in CNN such as ReLU or sigmoid, $\max_{x\in \mathbb{R}}\left(\frac{dh\left(x\right)}{dx}\right)\leq 1$ and $\min_{x\in \mathbb{R}}\left(\frac{dh\left(x\right)}{dx}\right)\geq 0$, and then:

(18)
$$\begin{array}{l} ℒ\left({ℱ}^{\left(l+1,n_{l+1}\right)},{ℱ}_{p}^{\left(l+1,n_{l+1}\right)}\right)\\ \leq \frac{1}{a_{l+1}}K^{2}{\left\|W^{\left(i,n_{l+1}\right)}\right\|}_{2}^{2}{\left\|\delta_{i,j}^{\left(l\right)}\right\|}_{2}^{2}\\ =\frac{1}{a_{l+1}}K^{2}{\left\|W^{\left(i,n_{l+1}\right)}\right\|}_{2}^{2}{\left\|h\left({ℱ}^{\left(l,i\right)}\right)-h\left({ℱ}^{\left(l,j\right)}\right)\right\|}_{2}^{2}\\ \leq \frac{1}{a_{l+1}}K^{2}{\left\|W^{\left(i,n_{l+1}\right)}\right\|}_{2}^{2}{\left\|{ℱ}^{\left(l,i\right)}-{ℱ}^{\left(l,j\right)}\right\|}_{2}^{2}\\ =\frac{a_{l}}{a_{l+1}}K^{2}{\left\|W^{\left(i,n_{l+1}\right)}\right\|}_{2}^{2}ℒ\left({ℱ}^{\left(l,i\right)},{ℱ}^{\left(l,j\right)}\right) \end{array}$$

Since $F^{\left(l,i\right)}$ is an arbitrary channel of the *l*_*th*_ layer, we can further narrow the upper bound:

(19)
$$\begin{array}{l} \begin{array}{l} ℒ\left({ℱ}^{\left(l+1,n_{l+1}\right)},{ℱ}_{p}^{\left(l+1,n_{l+1}\right)}\right)\\ \leq \frac{a_{l}}{a_{l+1}}K^{2}{\left\|W^{\left(i,n_{l+1}\right)}\right\|}_{2}^{2}\min_{j\in \left\{1,\ldots ,N_{l}\right\}}ℒ\left({ℱ}^{\left(l,i\right)},{ℱ}^{\left(l,j\right)}\right)\\ =\varepsilon \times \min_{j\in \left\{1,\ldots ,N_{l}\right\}}ℒ\left({ℱ}^{\left(l,i\right)},{ℱ}^{\left(l,j\right)}\right) \end{array} \end{array}$$

where $\varepsilon =\frac{a_{l}}{a_{l+1}}K^{2}||W^{\left(i,n_{l+1}\right)}|{|}_{2}^{2}$ and *a*_*l*_ = *H*_*l*_×*W*_*l*_×*B*.
